# Impact of cigarette price and tobacco control policies on youth smoking experimentation in Albania

**DOI:** 10.1136/tc-2023-058196

**Published:** 2024-03-05

**Authors:** Elvina Merkaj, Edvin Zhllima, Drini Imami, Irena Gjika, Carlos Manuel Guerrero-López, Jeffrey Drope

**Affiliations:** 1 Polytechnic University of Marche, Ancona, Italy; 2 Agricultural University of Tirana, Tirana, Albania; 3 Development Solutions Associates (DSA), Tirana, Albania; 4 Parliament of Albania, Tirana, Albania; 5 Johns Hopkins Bloomberg School of Public Health, Baltimore, Maryland, USA

**Keywords:** Price, Public policy, Economics, Taxation, Prevention

## Abstract

**Background:**

Albania has one of the highest smoking prevalence in Europe especially among the youth. There is a lack of evidence in Albania, as well as in most of Eastern Europe and middle-income countries, regarding the effect of price on smoking experimentation.

**Objective:**

The study aims to assess the effect of price and tobacco control policies on youth smoking experimentation in Albania.

**Methods:**

We used microdata from the Global Youth Tobacco Survey in Albania for 2004, 2009, 2015 and 2020. We constructed a pseudo-longitudinal dataset and estimated a split-population model to assess the hazard of smoking experimentation.

**Results:**

Price is a significant predictor of smoking experimentation among teenagers in Albania for both males and females (p<0.001). Being male increases the odds for smoking experimentation by more than 50% as compared with females (p<0.001), whereas females appear to be more price sensitive. Peer and parent smoking are also important determinants for smoking experimentation. Introducing penalties for smokers and legal entities violating smoke-free policies implemented in 2014 is also associated with a lower hazard of smoking experimentation.

**Conclusion:**

Price is a significant predictor of smoking experimentation among teenagers in Albania for both males and females. A combination of increasing taxes and strengthening the rule of law to control tobacco use in public spaces, in addition to public awareness campaigns targeting both youth and smoking parents, could help to significantly reduce the probability of smoking experimentation.

**WHAT IS ALREADY KNOWN ON THIS TOPIC**

- Taxes that lead to higher prices are associated with lower consumption and lower hazard of smoking experimentation.

**WHAT THIS STUDY ADDS**

- Although there are previous studies that examine smoking experimentation among youth, few examine the specific contexts of newer democracies and post-socialist countries under transition characterised by weak law enforcement as is the case of Albania, a country with one of the highest smoking prevalences in Europe.
- This research finds that consistent, with other contexts, higher price is associated with a lower hazard of smoking experimentation among youth in Albania.
- Females seem to be more price responsive than their male counterparts. Smoke-free policies have reduced smoking experimentation among youth.

**HOW THIS STUDY MIGHT AFFECT RESEARCH, PRACTICE OR POLICY**

- Increasing taxes on tobacco products that lead to higher prices could help to prevent the youth population in Albania from smoking experimentation and eventual initiation or at least delay them, while banning smoking in public spaces by implementing comprehensive legislation with strict monetary penalties could reinforce its protective effect towards this vulnerable group of population.

## Introduction

The earlier in life an individual tries smoking, the higher is the likelihood that he/she will become a regular smoker. There is substantial evidence that for some and even many young people, the first few cigarettes are enough to trigger a vulnerability to nicotine dependence.^1–3^ Other research shows that 30%–70% of experimenters eventually progress to dependence.^4–6^ Moreover, adolescents consistently underestimate the risk of becoming addicted to smoking and likely see little danger in experimenting.^6 7^ Therefore, to achieve lower smoking prevalence at a population level, it is crucial to tackle the issue of youth smoking.

Taxes on tobacco are associated with lower hazards of smoking experimentation and onset,^8–17^ especially among youth.^9^ Young smokers are generally more responsive to cigarette price changes than adults due to lower disposable income. Younger smokers also typically have lower levels of smoking addiction because of shorter smoking histories.^18 19^ Gender may condition the effect of price in the likelihood of trying and/or initiating regular smoking, although research has generated mixed results here.^17 18 20–22^

Research also shows that non-price tobacco control policies negatively affect tobacco experimentation and initiation.^8 23^ These policies include health warnings, minimum purchase ages and restrictions, marketing restrictions and smoke-free policies, among others.

Peer relationships are important factors in youth smoking.^16 24–28^ Smoking in the family is a significant determinant of youth smoking,^26 29 30^ although higher levels of family education and wealth have a negative effect.^17 31^

None of the predictors of youth smoking experimentation and/or initiation mentioned above have been explored yet in southeastern Europe. Indeed, studies have been limited only to a few sociodemographic characteristics,^32 33^ thus the effects of prices and other tobacco control instruments have not been adequately assessed.

Consequently, this research explores the main predictors for smoking experimentation among Albanian youth, with focus on both price and non-price tobacco control policies.

### Cigarette price and tobacco control policies in Albania

Albania is a Southern European emerging post-communist economy with a population of 2.8 million people. Tobacco use in Albania is especially high among youth and males^34^ and this is a major public health and economic concern^35 35^ as 15.3% of adolescents (ages 13–15 years old) use tobacco.^36^ The issue is critical considering the young population of Albania. Accordingly, mitigating youth smoking should be a priority for policymakers.

Excise tax increases on cigarettes that lead to price increases are instrumental for reducing tobacco consumption in Albania.^34^ There is a significant space to use fiscal policies to help discourage smoking considering that excise taxes in Albania are the lowest compared with all southeastern European countries, and the regional average itself falls well below that of the European Union.

**Figure 1:**
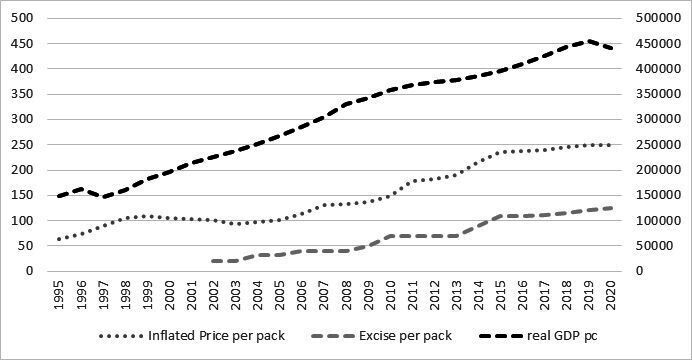
Trend of the price per pack of cigarettes, excise tax per pack and Gross Domestic Product (GDP) in Albanian Lek (ALL). Albania, 1995–2020. Sources: World Bank; National Institute of Statistics and General Directory of Customs in Albania.

Changes in excise taxes in Albania have been associated with increasing prices over the last 25 years; however, cigarettes remain affordable as Gross Domestic Product (GDP) per capita has also grown at a similar pace. Figure 1 illustrates the trend of the real retail price of cigarettes, excise tax and GDP.

In addition, the Government of Albania introduced several tobacco control policies during the early post-socialist transition all characterised by weak law enforcement, among others: a law that banned some tobacco advertisement on TV, radio, print media and billboards in 2001, and the law implemented in 2007 that prohibits all types of advertising, promotion, and sponsorship of tobacco on radio, TV, and print media; and establishes a minimum age for tobacco sale.

The Law No. 8691, “For the production and trade of tobacco and cigarettes,” entered into force in 2013 to control informal tobacco production, sales and advertising. This law included several measures such as nearly doubling the maximum fines. In 2014, the Law No. 76/2014 was introduced to better restrict tobacco use in indoor spaces. This law reasserted the obligation for the inclusion of educational programmes for the protection of health from tobacco products in TV and raised the maximum level of fines for smokers and managers/administrators of public or private entities in cases of violating the smoke-free environments.

We also examined the impact of tobacco control policies implemented in 2013 and 2014 which represent the first major government’s efforts to implement tobacco control laws since the fall of the communism.

### Data and analytical approach

The study analyses data from the GYTS implemented in Albania in 2004, 2009, 2015 and 2020.^36–39^ The GYTS is a cross-sectional, school-based survey on tobacco use among school children aged 11–17 years old, with a standardised methodology which is nationally representative, with a two-stage sample design. The survey is based on a structured questionnaire consisting of several topics such as children’s knowledge and attitudes towards smoking, prevalence of tobacco use, the role of advertising and media in tobacco use, and access to cigarettes. The Albanian GYTS administered in 2004 covered 4682 students, 4771 students in 2009, 4672 students in 2015 and 5388 students in 2020, for a total of 19 513 students interviewed.

Duration/survival analysis is used to estimate the relationship between tobacco control policies and smoking experimentation for youth. Survival analysis addresses the issue of incomplete information from individuals who may not have experimented with smoking before the end of the data collection.^17 31 40–46^ Observations from these individuals are right censored and excluding these individuals, as happens in standard methodologies, distorts the distribution of event duration.^17 47^

Standard survival models implicitly assume that all individuals under observation will eventually experience the event of interest (smoking experimentation).^17 19^ However, this is very restrictive for smoking onset, as a share of individuals may never experiment with smoking. To relax this assumption, and to treat right censoring, like previous research^15 17 29 32 40 44 48^ we use a discrete-time split population survival model to analyse the impact of price policies on smoking experimentation. The split population model is a discrete-time proportional hazard (cloglog) model, with an additional restriction that supposes a proportion of the population never fails (ie, experiments with smoking) and estimates the hazard rate for the remainder of the population that has a positive probability of smoking experimentation.^17 49^ Splitting the population in a group that will not fail and another group that will eventually fail—makes the model more accurate. However, we use also standard cloglog discrete hazard survival model as a sensitivity analysis.

More precisely, the (log) likelihood function to be maximised is as follows:

$$ln(L)=\begin{array}{l} \Sigma w_{i}c_{i}ln[\Phi (\alpha^{\prime }z_{i})f(t|s_{i}=1,x_{i}(t))]\;+(1-c_{i})ln[1-\Phi (\alpha^{\prime }z_{i})+\Phi (\alpha^{\prime }z_{i})S(t|s_{i}=1,x_{i}(t))] \end{array}$$

where $w_{i}$ =survey weights; $c_{i}$ =1 if *i* ever smoked; $s_{i}$ =1 if *i* will eventually start smoking and 0 if they never do; $z_{i}$ is time-invariant covariates; $x_{i}\left(t\right)$ is time-varying covariates including the price of cigarettes; $\Phi \left(\alpha^{\prime }z_{i}\right)=Pr\left(y_{i}=1|z_{i}\right)$ is the probability of smoking (probit); and $f(t|s_{i}=1,x_{i}\left(t\right))$ is the conditional density function of trying smoking at the observed starting age. Finally, $\Phi \left(\alpha^{\prime }z_{i}\right)S(t|s_{i}=1,x_{i}(t)$ is the probability of starting after the age observed in the survey.

One key assumption of survival models, known as non-informative censoring, is that survival and censoring are independent. To deal with this limitation, as reported elsewhere,^50^ we excluded younger respondents who are less likely to have experimented with smoking and more likely to be censored and focused on individuals aged 15–17 years at interview.

We organised the data in a pseudo-longitudinal format to analyse the effects of tobacco policies from 1994 to 2020. We infer the year of first smoking experimentation from the question, ‘How old were you when you first tried a cigarette?’. Knowing the respondent’s current age at the year of the interview, we can track the smoking status of the students for the entire period of analysis.

### Variables

The dependent variable is first smoking experimentation.^29^ The variable uses the answer to the question, ‘How old were you when you first tried a cigarette?’. The age of first experimentation with smoking in all GYTS was provided in 2-year intervals (such as 8–9 years old, 10–11 years old, etc). We randomly selected between the upper and the lower age of the interval if it was not higher than the current age during the interview year. Unfortunately, like all GYTS and most health surveys, no survey questions indicate when respondents become regular smokers.

The hazard of smoking experimentation is modelled as a function of cigarette prices, non-price tobacco control policies, gender, parental smoking behaviour and peer smoking behaviour. Our key variable of interest is the price of a 20-cigarette pack. GYTS does not include information on price of cigarettes, so we use the average deflated price of cigarettes for the period 2003–2017 as provided by the Albanian Institute of Statistics (INSTAT). For the period 1994–2002 and 2018–2020, we calculated the cigarette price from the yearly change in the Consumer Price Index for cigarettes as reported by INSTAT. An advantage of using mean price, instead of price by brand declared by the respondents, is that it rules out endogeneity between price and consumption, one of the major concerns to disentangle the effect of price as it can lead to biased estimates.^51^ A limitation of using average cigarette price is that it does not permit capturing price differences by brand at the time of initiation.^19^ Even if most/all the youth respondents had chosen to buy another cheaper or more expensive brand, it is unlikely to greatly influence the mean price of cigarettes because youth represent a small share of tobacco consumers.

By interacting the price variable with gender, we can estimate the impact of cigarette prices on smoking experimentation by gender. In this study, we use respondent-indicated sex as a proxy of gender.

We include two dichotomous indicators to capture the implementation of the two regulatory acts of 2013 and 2014. To control for income growth, we include a GDP per capita measure for each year.

We control for demographics including gender, and contextual factors including peer smoking habits and parental smoking behaviour (see online supplemental table A1). The main concern about these variables is endogeneity. We tried to mitigate endogeneity of peer behaviour by using the variable *‘*Most close friends smoke’ which is a dummy variable. However, we acknowledge that it is significantly difficult for an individual to influence the behaviour of most/all their peers. Similarly, we argue that it is difficult for a young person to influence greatly the smoking behaviour of a parent who likely initiated many years before the child was born.

10.1136/tc-2023-058196.supp1 Supplementary data

We assume that individuals are first exposed to the risk of smoking experimentation at the age of nine. It is generally not recommended to include a measure of calendar time in duration models.^52^ We used duration dependence, that is, how the hazard varies with survival time. We control for different forms of duration dependence in every model (see the online supplemental table A2). The analyses are performed with STATA V.17. The split population model is estimated through the spsurv command. The baseline models are chosen based on values of the Akaike information criterion (AIC) and Bayesian information criterion (BIC).

## Results

The number of individuals observed in the four waves is 6816. The number of experimenters is 2785 (1180 females and 1605 males). Just over 40.86% of respondents indicated that they had experimented with smoking cigarettes sometime during their lifetime.

The age of smoking experimentation indicates features of children’s smoking behaviours and exposure to tobacco in their early life. The average age those reporting smoking is 15.46 years old, with no significant difference between males and females (15.48 vs 15.45). The average age of smoking experimentation is 12.95 (13.16 for females vs 12.79 for males, with no statistically significant difference). Half of the smokers experimented with smoking before age 13, and 75% before age 15 years old. Only 1% of respondents tried smoking before 9 years old.

### Results of the split population model

Table 1 reports the estimates of the hazard of smoking experimentation from the split population and cloglog model. Model 1 is our baseline model, while model 2 presents the estimates of the sensitivity to the cigarette price by gender. The estimates were exponentiated and values below 1 are interpreted to reduce the hazard of smoking by the distance to 1, while those above 1 increase the hazard of smoking experimentation by the amount over 1.

**Table 1:** Split population survival model

Hazard of smoking experimentation	Split population model		Cloglog hazard model	
	Model 1	Model 2	Model 1/a	Model 2/a
Price of cigarettes	0.988^***^		0.990^***^	
	(0.002)		(0.002)	
Price*Female		0.986^***^		0.986^***^
		(0.002)		(0.002)
Price*Male		0.990**		0.990^***^
		(0.002)		(0.002)
Producers, traders, advertisers control policy	1.121	1.103	0.922	1.095
	(0.137)	(0.135)	(0.117)	(0.133)
Indoor smoking control policy	0.770**	0.752**	0.833^*^	0.759^***^
	(0.081)	(0.079)	(0.088)	(0.080)
Female	0.481^***^		0.500^***^	
	(0.021)		(0.020)	
A family member smokes in home premises	1.529^***^	1.503^***^	1.490^***^	1.476^***^
	(0.065)	(0.064)	(0.058)	(0.058)
Most of the peers smoke	1.690^***^	1.649^***^	1.654^***^	1.628^***^
	(0.081)	(0.078)	(0.072)	(0.071)
GDP per capita	1.000488^***^	1.00048^***^	1.000486^***^	1.000477^***^
	(0.00008)	(0.00008)	(0.00008)	(0.00008)
Duration dependence	Yes	Yes	Yes	Yes
Observations	55 291	55 291	55 291	55 291
Cure probability	0.088^***^	0.067^***^		

For the sake of interpretability, HRs are shown. SEs are in parentheses. Errors are clustered at the individual level in the cloglog hazard model. Spsurv command did not allow clustering of SEs. We control for duration dependence in both models. Our baseline models were chosen based on the values of AIC and BIC. Various robustness checks are presented in the online supplemental appendix.

*p<0.10; **p<0.05; ***p<0.01.

AIC, Akaike information criterion; BIC, Bayesian information criterion.

Model 1’s results suggest that price has a strong impact on the decision to experiment with smoking among teenagers in Albania. For every 1 Albanian Lek increase in the price of cigarettes, smoking experimentation is expected to decrease by 1.2% (ie, 1–0.988). In other words, a 10% increase in price (14.6 Lek) will reduce the hazard of smoking experimentation on average by almost 17% (14.6*1.2). Furthermore, gender is statistically significant also in model 1, suggesting that females are almost 50% less likely to experiment with smoking than males.

Price is a negative predictor of smoking for both males and females (see Model 2). Our results suggest that increments in cigarette prices reduce the probability of smoking experimentation for girls by a greater percentage than for boys (p value<0.001). An increase of 10% in price would reduce on average the likelihood of smoking by almost 20% among females compared with 14% among males.

Smoke-free policies introduced in 2014 appear to have reduced experimentation by 23%. This effect is also likely partially explained by a possible time-lagged effect of the 2013 tobacco control policies. On the other hand, GDP per capita has also a statistically significant impact on the experimentation with smoking. An increase of the real GDP per capita by 5% increases the hazard of smoking by 7.4%.

Due to the potential endogeneity concerns highlighted above, we are cautious not to overinterpret the effect size of the peers and family smoking coefficients. We find a high correlation between the hazard of smoking experimentation in youth and both peer and parental smoking behaviour. Even when we use different variables to measure peer and family smoking patterns, the association remains strong. These variables are impactful but further research is needed to disentangle their effects on smoking experimentation.

### Robustness checks

Various tests were conducted to check the robustness of the results (see online supplemental tables A2–A4).

The main results are robust to different specifications. We first include in the regression only price, then add the control variables one by one to see if the effect of any control variable would influence the size of our focus variables. Results show the robustness of the price and policies effect. Given the potential endogeneity of the peer and parental control variables, we employ different variables to measure the importance of the family and peer smoking behaviour. We replace the variable ‘A family member smokes in home premises’ with variables such as ‘At least one parent smoke’. The variable ‘most/ all of peers’ smoke’ is replaced with the variable ‘Most/all of the close friends smoke’. The correlation of peers and family smoking with youth experimentation behaviour remains significant, and the main results did not change, confirming the robustness of the baseline estimates.

The GDP with local current unit, and the GDP growth were used as a sensitivity check. Different time dependences, up to the fourth polynomial degree, are tested. All estimates were also performed by a standard discrete-time hazard cloglog survival model, which generated similar results, confirming the robustness of our main results.

## Discussion

All the models suggest that price has a negative and significant relationship with smoking experimentation in Albania, with a magnitude similar to other scholars’ findings.^29 44 52^

The results are in line with some studies which show that female youth are more sensitive to price increase (eg, in terms of initiation),^15 21 22^ thus are in contrast with other studies which observe that higher cigarette prices decrease the probability of smoking initiation among males but have no impact on female smoking initiation as compared with males.^20^ However, similar to findings from other scholars,^10^ the demand for tobacco by males appears less price elastic than the demand for cigarettes by females. Being male also increases the odds for smoking experimentation by more than 50%.^53^ This is similar to other empirical findings on gender differences in smoking experimentation.^52^

Tobacco control policies have a positive role in reducing smoking experimentation in Albania. Similar to findings from other researchers,^17 19 40^ we found that smoke-free policies have a significant negative effect on smoking experimentation.

Peer and family smoking appear to have substantial effects on youth smoking experimentation. Teenagers that live in smoking environments with friends, classmates and/or parents who smoke are more likely to try smoking. These findings are similar to other research, adding to evidence that tobacco use may be transmitted intergenerationally.^26 29 30^

The study has several limitations. Few variables related to access to anti-tobacco education at schools, tobacco advertising, financial resources allocated to smoking, family wealth and parents’ education were available due to the inconsistent structure of questions among the GYTS rounds. Difficulty controlling for unobserved characteristics of the network/environment or socioeconomic situation of the family/school might generate endogeneity of the peer and family variables. We note that this is a limitation of our study, but also emphasise that estimating these effects is not the aim of our analyses and we include them instead as controls. We also try specifications including one or neither of these variables and find that the effects of price and tobacco control policies on tobacco expenditures, the study’s focus, remain consistent. We acknowledge that, even though we try to mitigate informative censoring and endogeneity issues, we cannot rule out the possibility that our analysis may still face these issues. However, despite these limitations the study captures most tobacco experimentation predictors and provides strong evidence supporting tobacco control policies—including excise taxation—in Albania.

Finally, we use experimentation and not initiation of regular smoking as the dependent variable. It is a related but different query to identify and understand the variables that drive initiation. If the data had existed, we would have examined this important relationship. But one might reasonably argue that we would expect an even larger effect of price on initiation because it costs more to be a regular smoker than an experimenter. Thus, the fact that this research finds a strong relationship between price and experimentation suggests that future research should try to examine initiation more closely if the data become available to explore this hypothesis.

## Conclusions

This research contributes to the scarce existing literature in Eastern Europe and low and middle income countries (LMICs) on youth smoking experimentation with a focus on price. The research finds that price is a key predictor of youth smoking experimentation in Albania. These findings suggest strongly that further tax increases that lead to price increases are likely to contribute to a greater decline in smoking rates and decrease smoking experimentation among youth. Even if an excise tax increase results only in a delay of smoking experimentation rather than permanent abstinence, it is still likely to have positive health effects because those individuals are less likely to become regular smokers over their lifetimes.^52^

We also find that smoke-free laws help to reduce smoking initiation.^54^ The law implemented in 2013 and 2014 had high deterrent effects. To reinforce the seriousness of enforcement, the government fined the most famous bars/restaurants substantially for violations, broadcasting their efforts publicly through the media.^54^ Future research could evaluate the impact of fines by experimentation methods, field observations and/or other qualitative approaches for exploring the behaviours of offenders.^55–57^

Our results also suggest that other variables such as gender, and parents’ and peers’ smoking, significantly affect smoking experimentation. Population-level awareness and education campaigns to address parents’ behaviours, particularly by pointing to their importance as role-models for their children and the home as a positive, educative environment, is likely to be a useful intervention for preventing or delaying smoking experimentation.

Furthermore, the findings show that income, measured through real GDP per capita, have a significant impact on smoking experimentation. This implies that it is crucial to consider GDP (income) growth when designing tobacco taxation so that higher tobacco excise can mitigate the positive effect of higher income.

## Data Availability

The main data used in this study is publicly available at: https://www.who.int/teams/noncommunicable-diseases/surveillance/data/albania.
