# Supplementary material for: Impact of cigarette price and tobacco control policies on youth smoking experimentation in Albania
Source: Tob Control. 2024 Mar 5;33(Suppl 2):s38–43. doi: 10.1136/tc-2023-058196 (PMC11187397; doi:10.1136/tc-2023-058196)
Supplement: Supplementary data [file tc-2023-058196supp001.pdf]

APPENDIX

Table A1 – Descriptive statistics according to year of implementation of variables

Variable name	Definition	GYTS 2004	GYTS 2009	GYTS 2015	GYTS 2020	Total sample
Cigarette price*	Real price per pack (inflation-adjusted) (in ALL)	97.8	136.6	235.5	250.4	145.99
**Tobacco control policies**						
Producers, traders, advertisers control policy	Dummy that takes value 1 after the year 2013, when the policy was implemented	0	0	1	1	0.30
Indoor smoking control policy	Dummy that takes value 1 after the year 2014, when the policy was implemented	0	0	1	1	0.24
**Control variables**						
Gender	Dummy that indicates when a respondent is female (in %)	0.53	0.52	0.51	0.51	0.52
One Parent smokes	Dummy that indicates if one of the respondent's parents (either of both) smoke	0.51	0.45	0.45	0.43	0.46
A family member smokes in home premises	Dummy that indicates when at least a family member smokes in home premises (mother, father, brother, sister)	0.50	0.45	0.45	0.42	0.45
Most Close Friends smoke	Dummy that indicates if most or all of respondent's closest friends' smoke tobacco	0.04	0.09	0.09	0.06	0.07
Most Students in my grade smoke	Dummy that indicates if at least half students in the respondent's grade smoke tobacco (half or all)	0.10	0.18	0.17	0.11	0.14
GDP per capita	The per capita GDP in 2015 constant prices, in ALL (source: World Bank) (1USD=100ALL)	252200	343200	395300	441100	303300

*Note: The price values included in the table are the average prices for the years in which GYTS were collected. Price values for all the period from 1994 to 2020 were included in the analyses.

Table A2: Sensitivity analysis: preliminary analysis

	Split population model						cloglog					
	m1	m2	m3	m4	m5	baseline model	m1	m2	m3	m4	m5	baseline model
price_female	0.991***	0.994***	0.987***	0.985***	0.987***	0.986***	0.992***	0.995***	0.987***	0.986***	0.987***	0.986***
	(0.000)	(0.001)	(0.002)	(0.002)	(0.002)	(0.002)	(0.000)	(0.001)	(0.002)	(0.002)	(0.002)	(0.002)
price_male	0.995***	0.999*	0.991***	0.990***	0.991***	0.990***	0.996***	0.999	0.991***	0.990***	0.991***	0.990***
	(0.000)	(0.001)	(0.002)	(0.002)	(0.001)	(0.002)	(0.000)	(0.001)	(0.002)	(0.002)	(0.002)	(0.002)
policy_2013		0.816*	1.063	1.117	1.057	1.103		0.783**	1.046	1.091	1.054	1.095
		(0.090)	(0.128)	(0.136)	(0.128)	(0.135)		(0.084)	(0.125)	(0.132)	(0.127)	(0.133)
policy_2014		0.694***	0.718***	0.710***	0.753***	0.752***		0.708***	0.731***	0.731***	0.757***	0.759***
		(0.072)	(0.074)	(0.075)	(0.078)	(0.079)		(0.072)	(0.075)	(0.076)	(0.078)	(0.080)
gdp_pc_const2015			1.000***	1.001***	1.000***	1.000***			1.000***	1.001***	1.000***	1.000***
			(0.000)	(0.000)	(0.000)	(0.000)			(0.000)	(0.000)	(0.000)	(0.000)
smosmokehome_dummy				1.581***		1.503***				1.495***		1.476***
				(0.072)		(0.064)				(0.058)		(0.058)
st_grade_smoke_mosthalf					1.652***	1.649***					1.639***	1.628***
					(0.077)	(0.078)					(0.071)	(0.071)
Duration dependence	Yes	Yes	Yes	Yes	Yes	Yes	Yes	Yes	Yes	Yes	Yes	Yes
Observations	56863	56863	56863	55755	56374	55291	56863	56863	56863	55755	56374	55291
AIC	19646	19618	19587	19092	19318	18854	19656.64	19623.9	19589.56	19100.54	19316.89	18854.31
BIC	19691	19681	19658	19173	19399	18943	19692.43	19677.59	19652.2	19171.97	19388.41	18934.6
curep	0.204	0.172	0.145	0.174	0.0357	0.0667						

Table A3: Sensitivity analysis: Different control variables using Split population model

Hazard of smoking	Baseline model-alternative time dependence variable	Baseline model-alternative peer and parental smoking variables	Baseline model-Alternative GDP variable	Baseline model-Alternative GDP variable	Alternative GDP variable-alternative peer and parental smoking variables	Alternative GDP-alternative time dependence
the price calculated by us	0.989***	0.989***	0.987***		0.988***	0.988***
	-0.002	-0.001	-0.002		-0.002	-0.002
price_female				0.985***		
				-0.002		
price_male				0.989***		
				-0.002		
policy_2013	0.99	1.075	1.125	1.107	1.078	0.992
	-0.123	-0.13	-0.139	-0.137	-0.131	-0.124
policy_2014	0.751***	0.762***	0.786**	0.768**	0.777**	0.765**
	-0.08	-0.078	-0.083	-0.081	-0.08	-0.082
0-male 1-female	0.446***	0.529***	0.481***		0.529***	0.445***
	-0.021	-0.021	-0.021		-0.022	-0.021
smosmokehome_dummy	1.610***		1.529***	1.503***		1.610***
	-0.074		-0.065	-0.064		-0.074
st_grade_smoke_mosthalf	1.687***		1.692***	1.651***		1.687***
	-0.089		-0.081	-0.078		-0.089
At least one parent smokes		1.153***			1.153***	
		-0.045			-0.045	
friends_smoke_mostall		2.200***			2.202***	
		-0.117			-0.117	
gdp_pc_const2015	1.000***	1.000***				
	0	0				
gdp_pc_lcu			1.000***	1.000***	1.000***	1.000***
			0	0	0	0
logt		7.613***	8.118***	7.794***	7.679***	
		-0.469	-0.568	-0.556	-0.475	
t	1.511***					1.514***
	-0.019					-0.02
Observations	55291	56275	55291	55291	56275	55291
AIC	18993.18	19141.06	18796.56	18857.72	19144.34	18996.19
BIC	19082.38	19230.44	18885.77	18946.92	19233.72	19085.39
curep	0.218	0.00469	0.0909	0.0702	0.00549	0.221

Table A4: Sensitivity analysis: Different control variables using cloglog discrete time hazard survival model

Over14, cloglog	Baseline model-alternative peer and parental smoking variables	Baseline model-alternative time dependence variable	Baseline model-alternative time dependence variable	Baseline model-Alternative GDP variable	Baseline model-Alternative GDP variable	Alternative GDP variable-alternative peer and parental smoking variables	Alternative GDP-alternative time dependence	Alternative GDP-alternative time dependence
the price calculated by us	0.989***	0.989***	0.990***	0.987***		0.988***	0.988***	0.990***
	-0.002	-0.002	-0.002	-0.002		-0.002	-0.002	-0.002
price_female					0.985***			
					-0.002			
price_male					0.989***			
					-0.002			
policy_2013	1.075	0.993	0.922	1.113	1.098	1.077	0.991	0.913
	-0.129	-0.123	-0.117	-0.136	-0.134	-0.13	-0.124	-0.117
policy_2014	0.763***	0.778**	0.833*	0.794**	0.775**	0.778**	0.793**	0.846
	-0.078	-0.082	-0.088	-0.083	-0.082	-0.08	-0.083	-0.089
0-male 1-female	0.530***	0.500***	0.500***	0.501***		0.530***	0.501***	0.501***
	-0.02	-0.02	-0.019	-0.02		-0.02	-0.02	-0.019
smosmokehome_dummy		1.486***	1.490***	1.485***	1.475***		1.484***	1.489***
		-0.059	-0.058	-0.058	-0.058		-0.059	-0.058
st_grade_smoke_mosthalf		1.661***	1.654***	1.658***	1.630***		1.663***	1.657***
		-0.074	-0.072	-0.073	-0.071		-0.074	-0.072
At least one parent smokes	1.152***					1.152***		
	-0.044					-0.044		
friends_smoke_mostall	2.195***					2.196***		
gdp_pc_const2015	1.000***	1.000***	1.000***					
	0	0	0					
gdp per capita current lcu				1.000***	1.000***	1.000***	1.000***	1.000***
				0	0	0	0	0
logt	7.579***			7.394***	7.326***	7.638***		
	-0.38			-0.37	-0.365	-0.384		
	-0.108					-0.108		
t		1.427***	12606.9***				1.429***	11905.8***
		-0.012	-8949.974				-0.012	-8478.508
t2			0.0973***					0.0988***

			-0.019					-0.02
t3			1.286***					1.284***
			-0.03					-0.03
t4			0.990***					0.990***
			-0.001					-0.001
Observations	56275	55291	55291	55291	55291	56275	55291	55291
AIC	19139.09	19029.35	18657.53	18799.31	18857.6	19142.4	19033.6	18663.15
BIC	19219.53	19109.63	18764.58	18879.59	18937.9	19222.8	19113.9	18770.2
